# Anatomical Description of the Parts Concerned in Inguinal and Femoral Hernia. Translated from the French of M. Jules Cloquet; with Lithographic Plates from the Original Etchings, and a Few Additional Explanatory Notes

**Published:** 1835-04-01

**Authors:** 


*Anatomical Description of the Parts concerned in Inguinal and Femoral Hernia. Translated from the French of* M. Jules Cloquet; *with Lithographic Plates from the original Etchings, and a few additional Explanatory Notes.* By Andrew Melville M'Whinnie, Assistant Teacher of Practical Anatomy at St. Bartholomew's Hospital.—London, 1835. Royal 8vo. pp. 50.

This is a useful book, and, though perhaps scarcely minute enough in some parts for modern wants, should yet be carefully perused by the surgical student. The following quotations may give some notion of its manner.

"The fascia transversalis, then, is an aponeurosis, which, varying* in thickness, arises from the posterior edge of Poupart's ligament, from the fascia iliaca, from the external edge of the tendon of the rectus muscle, and is continuous above with the cellular tissue situated upon the internal surface of the abdominal muscles; inferiorly, and towards the middle of the crural arch, it forms a membranous canal, which commences by a wide opening, directed posteriorly and externally, and of which the internal edge is much the stronger: this canal descends around the spermatic vessels, to compose their proper sheath. The fascia transversalis supports the peritoneum behind, and is separated from it by the epigastric artery;† in front it corresponds with the transversalis muscle, and with the aponeurosis, of which it is often so intimately connected, that it can only be distinguished from it by the different direction of its fibres." (P. 16.)

"The inguinal canal is wider, and its apertures are much more distinct in the male than in the female. Its direction, which usually corresponds with that of the crural arch, is also a little more oblique in the former than in the latter.‡ The occurrence of inguinal hernia is influenced by the differences in the dimensions of the inguinal canal, and which depend on the age and sex. In the numerous measurements which I have made of the parts which relate to this subject, I have obtained very nearly the same results

* "The fascia transversalis is in some individuals extremely thin; in others, towards the rectus muscle, it is composed of very strong bundles of fibres, so disposed, that open spaces, varying in form and number, are left between them.

† "The fascia transversalis possesses the greatest strength between the superior opening of the inguinal canal and the rectus muscle. In this situation it is opposite to the posterior part of the inguinal ring, from which it is separated merely by the thin fibres of the internal oblique and transversalis, which are attached to the pubes.

‡ "If a horizontal line be drawn on a level with the pubes, and an oblique one from the symphysis pubis to the anterior superior spine of the ilium, it will be found that, in the female, these two lines will meet at a more acute angle than in the male, and which depends on the less degree of elevation and the greater breadth of the pelvis in the former: in the female, also, the crural arch is more horizontal. This difference, however, is not striking in many individuals."

as those published by Astley Cooper, in the second part of his Treatise on Hernia.

| | MALE. Inches. | FEMALE. Inches. |
|---|---|---|
| "From the symphisis pubis | | |
| to the anterior superior spine of the ilium . . . | $5\frac{3}{4}$ | 6 |
| to the tuberosity of the pubes . . . . . . . . . | $1\frac{1}{8}$ | $1\frac{3}{8}$ |
| to the inner margin of the external abdominal ring | $0\frac{7}{8}$ | 1 |
| to the inner margin of the superior aperture of the inguinal canal . . . . . . . . . . . . . | 3 | $3\frac{1}{4}$ |
| to the middle of the iliac artery . . . . . . . | $3\frac{1}{8}$ | $3\frac{3}{8}$ |
| to the middle of the iliac vein . . . . . . . . | $2\frac{5}{8}$ | $2\frac{3}{4}$ |
| to the origin of the epigastric artery . . . . . | 3 | $3\frac{1}{4}$ |
| to the point where the epigastric artery passes on the inner side of the superior opening of the inguinal canal . . . . . . . . . . . . | $2\frac{3}{4}$ | $2\frac{7}{8}$" |

(P. 22.)

Cloquet says,—

"The crural canal presents three walls: the anterior extends from the crural arch to the upper part of the opening for the vena saphena, (Plate II. 4,) and is formed by the superficial layer of the fascia lata, which ascends in front of the femoral vessels; it is much stronger externally than on the inner side, where it is continuous with the deep layer of the same fascia, and with Gimbernat's ligament." (P. 36.)

His translator adds,

"The anterior boundary or wall of the crural canal is described by Sir Astley Cooper as being derived from the fascia transversalis, which is continued, he says, downwards beneath Poupart's ligament. It has been named Fascia Cribrosa, in consequence of its being perforated by small holes for the passage of blood-vessels and lymphatics. See Plate II. 4.—(P. 36, *note.*)

On this point we rather agree with Sir Astley than with the French anatomist, and are consequently of opinion that the plate in which the latter has embodied his description is not conformable to nature.

